# Antiepileptic Effects of *Acorus tatarinowii* Schott in a Rat Model of Epilepsy: Regulation of Metabolic Axes and Gut Microbiota

**DOI:** 10.3390/biology14050488

**Published:** 2025-04-29

**Authors:** Liang Chen, Jiaxin Li, Wenhui Zhang, Jiepeng Wang

**Affiliations:** 1 School of Pharmacy, Guizhou University of Traditional Chinese Medicine, Guiyang 550025, China; chenliang029@gzy.edu.cn (L.C.); lijiaxin048@gzy.edu.cn (J.L.); zhangwenhui217@gzy.edu.cn (W.Z.); 2 School of Basic Medical Sciences, Hebei University of Chinese Medicine, Shijiazhuang 050200, China

**Keywords:** *Acorus tatarinowii* Schott, metabolomics, microbiota, epilepsy

## Abstract

As a phytotherapeutic agent with historical applications in epilepsy management, *Acorus tatarinowii* Schott (ATS) remains pharmacologically enigmatic, particularly regarding its pathophysiological mechanisms. This knowledge gap significantly hinders the clinical application of ATS-based treatments. To explore the potential of ATS in combating epileptogenesis, we utilized a pentylenetetrazole (PTZ)-induced chronic epilepsy rat model. Brain metabolomic analysis was performed by ultra-performance liquid chromatography coupled with mass spectrometry (UPLC/MS). Principal component analysis (PCA) and orthogonal projections to latent structures-discriminant analysis (OPLS-DA) were performed for screening differential metabolites. Gut microbiota composition was analyzed through 16S rRNA gene sequencing and examined using Spearman correlation analysis. The results show that oral ATS (50 mg/kg) significantly improved the seizure latency and pathology of rats with epilepsy. Ascorbate and aldarate metabolism, glycerophospholipid metabolism, arachidonic acid metabolism, and intestinal flora were crucial for ATS’s ability to counteract epilepsy. The therapeutic effects of ATS against epilepsy were investigated with brain metabolomics and gut microbiota analysis, providing the basis for further comprehensive research.

## Simple Summary

*Acorus tatarinowii* Schott (ATS) is a plant-based medicine that has been used historically to manage epilepsy, but its exact mechanisms are still not fully understood. This lack of clarity limits its use in clinical treatments. To explore how ATS might help prevent epilepsy, we used a rat model with chronic epilepsy induced by pentylenetetrazole (PTZ). We analyzed the brain’s chemical makeup using a technique called UPLC/MS and identified key differences with statistical methods like PCA and OPLS-DA. We also looked at the gut bacteria using 16S rRNA sequencing. Our results showed that giving ATS orally (50 mg/kg) significantly improved seizure latency and pathology in epileptic rats. Important factors included changes in ascorbate and aldarate metabolism, glycerophospholipid metabolism, arachidonic acid metabolism, and gut flora. These findings provide a foundation for further research into ATS’s potential as an epilepsy treatment.

## 1. Introduction

Epilepsy is a chronic disorder characterized by recurrent seizures due to sudden abnormal neuronal discharges, which can lead to a range of issues, including disturbances in consciousness, sensation, movement, behavior, mental state, and autonomic function [1]. Globally, epilepsy affects approximately 70 million individuals, with China having the highest number of epilepsy patients [2,3]. Epilepsy, with its complex causes and unclear mechanisms, stands as one of the most challenging diseases to treat in modern medicine [4]. Currently, the treatment and prevention of epilepsy mainly include drug therapy, surgical treatment, neuromodulation therapy, and dietary adjustment [5]. However, while chemical drugs bring good therapeutic effects, they are also accompanied by many adverse reactions of physical and mental injuries [6,7]. It has been documented that certain chemical antiepileptic drugs may exacerbate seizures in some epilepsy patients [8]. With the continuous deepening of research on epilepsy, traditional Chinese medicine has achieved good results in treating epilepsy in recent years, opening up new avenues for the treatment of epilepsy [9].

*Acorus tatarinowii* Schott (ATS) first appeared as a traditional Chinese medicine in the earliest Chinese medicinal classic work Shennong’s Classic of Materia Medica (written more than 2000 years ago during the Han Dynasty) [10]. In clinical practice, it has a long history of being commonly used to treat mental epilepsy, forgetfulness, insomnia, tinnitus, and deafness [11,12,13,14]. So far, various compounds have been obtained from ATS, mainly including volatile oils, organic acids, terpenes, flavonoids, and other chemical components [15]. Research has shown that ATS has a definite antiepileptic effect, but its antiepileptic mechanism is not yet clear, which restricts its further development and utilization.

The gut microbiota is known as the second genome of humans, participating in a series of physiological and pathological processes, including those related to the central nervous system [16]. The communication between the gut microbiota and the brain is achieved through a complex bidirectional connection of the microbiota–gut–brain axis [17]. The gut microbiota can produce a series of important neurotransmitters during digestion and metabolism processes [18]. Recent research has indicated that intestinal dysfunction and ecological imbalance may be associated with the pathogenesis of and susceptibility to epilepsy [19,20]. When the microbial population becomes imbalanced, unhealthy signals are transmitted to the brain, resulting in adverse conditions such as increased oxidative stress [21,22]. There was evidence to suggest that dysbiosis of the gut microbiota was also related to changes in neuromodulators and disruption of the blood–brain barrier [23,24,25,26]. In other words, disruption of gut microbiota may be closely related to seizures and the effects of antiepileptic drugs [27,28].

As a transformative approach in systems biology, metabolomics provides unprecedented capacity for deciphering pathophysiological trajectories through dynamic monitoring of endogenous biomarker networks, offering metabolic fingerprints for the mechanistic elucidation of pharmacological interventions [29,30]. While seminal investigations have mapped metabolic perturbations in neuropsychiatric disorders—as evidenced by dysregulated neurotransmitter circuits in depression [31], amyloid-associated lipidome alterations in Alzheimer’s disease [32], and mitochondrial bioenergetic deficits in Parkinson’s disease [33]—the pharmacometabolomic interplay between *Acorus tatarinowii* Schott (ATS) and epileptogenesis remains conspicuously underexplored. Particularly noteworthy is the knowledge void regarding ATS’s bidirectional regulation of the gut–microbiota–brain axis during ictogenesis, including its potential to remodel microbial-derived epileptogenic metabolites.

Given the intricate interplay between epilepsy, gut microbiota, and brain metabolites, and the emerging potential of traditional Chinese medicine in treating epilepsy, this study aims to elucidate the therapeutic mechanisms of ATS in epilepsy treatment. This study will provide a comprehensive understanding of the pharmacological mechanisms underlying ATS, and offer insights into the development of novel therapeutic strategies targeting the gut–microbiota–brain axis for epilepsy treatment.

## 2. Materials and Methods

### 2.1. Chemicals and Drugs

Sodium valproate (SV) was obtained from Hunan Xiangzhong Pharmaceutical Co., Ltd. (Changsha, China,1H220403). Pentylenetetrazol (PTZ) was purchased from Aladdin Co., Ltd. (Shanghai, China, F2218455). KOD OneTM PCR Master Mix PCR enzyme and KOD FX Neo (TOYOBO) PCR enzyme were bought from Beijing Bailingke Biotechnology Co., Ltd (Beijing, China). Monarch DNA Gel Recovery Kit was purchased from Beijing Hongyue Innovation Technology Co., Ltd (Beijing, China). HPLC-grade acetonitrile and methanol were obtained from Concord Technology (Tianjin, China). All other reagents were of analytical grade, and doubly distilled water was used throughout the study.

*Acorus tatarinowii* Schott (ATS) was purchased from Kangmei Pharmaceutical Co., Ltd. (Shenzhen, China, 210702451). ATS was carefully weighed and soaked in 9 times the amount of distilled water for 4 h, then extracted with steam distillation for 6 h to obtain a thick yellow oil-like substance, that is, volatile oil. The volatile oil from ATS was dehydrated with anhydrous sodium sulfate and stored in 4 °C sealed condition for use.

### 2.2. Experimental Design

Male Sprague Dawley (SD) rats, aged 8 weeks and weighing 200 ± 20 g, were housed in a specific-pathogen-free (SPF) facility. This environment was controlled at a temperature of 22 ± 2 °C and a relative humidity of 55 ± 5%, and featured a 12 h light/12 h dark cycle. The study was approved by the Animal Care Welfare Committee of Guizhou University of Traditional Chinese Medicine (protocol code 20230225 and dated 24 October 2023). To ensure adequate adaptation and acclimatization to the laboratory environment, the SD rats were housed at the research center for 7 days prior to the commencement of the experiment. Throughout the study, the animals were provided with standard laboratory chow and free access to water. Seizure was induced by an intraperitoneal injection of PTZ (35 mg/kg body weight) to SD rats on alternate days for 30 days. In the control group, six male rats were administered an identical volume of saline solution. The behavioral manifestations of the animals during seizure episodes were assessed using a modified version of the Racine scale, as detailed in prior work [34]. The model was considered successful if the rats were subjected to more than 3 consecutive episodes of Racine V (facial clonus, rhythmic nodding, forelimb clonus, hind limb standing, and falling). The epileptic rats were randomly assigned to four groups: the model group (*n* = 6), the SV group (positive medicine, 100 mg/kg, *n* = 6), the ATS low-dose group (ATS-L, 10 mg/kg, *n* = 6), and the ATS high-dose group (ATS-H, 50 mg/kg, *n* = 6). Each group received oral administration of the respective treatment continuously for 4 weeks. For Western blotting, brain tissue protein was measured by Bradford assay, separated via SDS-PAGE, and transferred to a nitrocellulose membrane. After incubation with secondary antibody, immunoreactive bands were detected and their densities analyzed using ImageJ software (version 1.6).

### 2.3. Sample Collections and Preparation

After the rats were sacrificed, brain samples were collected from the SD rats. Brain tissues were collected in duplicate for each sample, one fixed in 4% paraformaldehyde for Nissl staining, and the other 50 mg of brain tissue sample was added to 1000 μL of extraction solution (methanol: acetonitrile: water, 2:2:1) and vortexed for 30 s. The brain homogenate was centrifuged in 4 °C and the supernatant was collected for further detection of metabolomics. The cecal contents from the rats were gathered and preserved in sterile containers (−80 °C), awaiting 16S rRNA gene sequencing analysis.

### 2.4. Metabolomics Analysis

To verify the accuracy of this method, 10 µL of each brain sample was collected and pooled to create quality control (QC) samples. All brain samples were run on a Xevo G2-XS ultra-performance liquid chromatography coupled with mass spectrometry (UPLC/MS) system (Waters, Milford, MA, USA). Samples were injected into Acquity UPLC HSS T3 (2.1 mm × 100 mm, 1.8 µm) and eluted with 0.1% formic acid (A) and acetonitrile (B) in a gradient at a flow rate of 0.40 mL min^−1^ for 15 min. The ms/ms spectrometry scans were in the range of *m*/*z* 50–1200. Electrospray ionization (ESI) ion source settings: capillary voltage 2500 V (positive mode) or −2000 V (negative mode) and cone-hole voltage 30 V.

Mass spectral raw data were processed in Progenesis Qi (Waters, Milford, MA, USA) for peak extraction, alignment, and normalization. PCA and OPLS-DA were conducted using R’s prcomp (version 3.6.1) and ropls (version 1.6.2). Differential metabolites were screened by Variable Importance in Projection (VIP) ≥ 1 and values with *p* < 0.05 using the T test. The Progression QI software (version 2024.0.1) online METLIN database and public database (HMDB) were used to identify the selected different metabolites and to enrich the metabolic pathway through the database KEGG (https://www.kegg.jp/kegg/pathway.html (accessed on 27 March 2024)) [35].

### 2.5. 16S rRNA Gene Sequencing Analysis

DNA concentration and purity were measured using the NanoDrop 2000 (Thermo Scientific, Waltham, MA, USA). The primers 27F2_(5’-AGRGTTTGATYNTGTCAG-3) and 1492R_(5’-TASGGHTACTTGTTASGACTT-3’) were added for 16S RNA full-length PCR amplification. The PCR products were purified, quantified, and homogenized to create a SMRT Bell sequencing library. This library was then bound to the primer and polymerase using the PacBio Binding kit. The final product was purified with AMpure PB Beams and sequenced on a Sequel II platform [36].

### 2.6. Statistical Analysis

The dates were represented by mean ± SEM. GraphPad Prism software (GraphPad, version 9.0) was used for the data analysis. The one-way ANOVA test was used for more than two groups and the non-parametric test was used for non-normal distribution. The correlation between the microbiome and brain metabolome was analyzed using the Spearman correlation tests.

## 3. Results

### 3.1. Therapeutic Efect of ATS on PTZ-Induced Epilepsy

**Figure 1:**
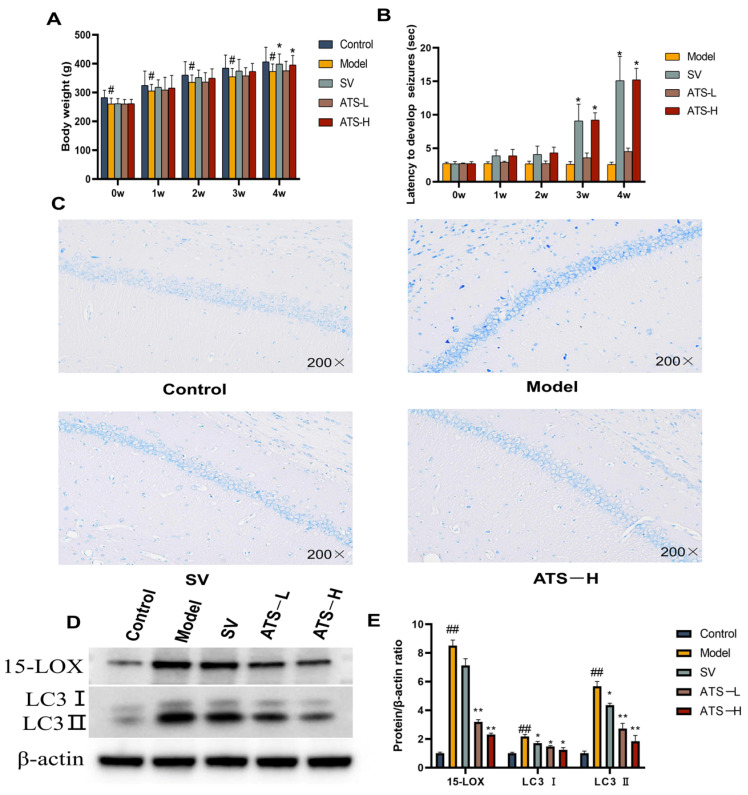
Effects of ATS on epilepsy in PTZ-induced epileptic rats. (**A**) The body weight of different groups of rats was assessed. (**B**) The latency to develop seizures. (**C**) Representative images of Nissl staining of the brains of rats from control, model, SV, and ATS-H (50 mg/kg) groups. (**D**,**E**) Expression of 15-LOX and LC3 was determined by Western blotting (Figures S1–S3). # *p* < 0.05, ## *p* < 0.01 vs. control group, * *p* < 0.05, ** *p* < 0.01 vs. model group.

In order to investigate the effect of ATS on the intensity of epileptic seizures, a chronic epilepsy rat model was established by intraperitoneal injection of PTZ. The results showed that rats treated with 50 mg/kg ATS had a significant increase in body weight compared to the model group in the fourth week (Figure 1A). Regarding seizure latency, rats treated with sodium valproate (SV) or 50 mg/kg ATS exhibited significantly longer latency than the PTZ-pretreated group (*p* < 0.05) (Figure 1B). However, no significant differences were observed between the low-ATS-dose group and the model group.

At 4 weeks following the administration of PTZ, the architecture of the hippocampus was evaluated in the experimental group using Nissl staining. Compared with the control group, the model group rats showed a small amount of nerve cell damage in the hippocampal tissue, and the cell bodies were condensed and darkened in color. Compared with the model group, the SV group of rats had a regular arrangement of hippocampal nerve cells, with cell body consolidation accompanied by a small amount of diffuse ablation of nerve cells. The tissue structure of ATS group rats is clear and the arrangement of hippocampal nerve cells is regular, and obvious inflammatory infiltration or morphological changes were not observed (Figure 1C). The results showed that PTZ-induced neuronal damage occurred in epileptic rats, and administration of ATS had a certain improvement effect.

To investigate the effects of ATS on epileptic seizures in rats, we measured the protein expression of epilepsy regulators (15-LOX, LC3 Ⅰ, and LC3 Ⅱ) by Western blotting. The expression of 15-LOX, LC3 Ⅰ, and LC3 Ⅱ in brain tissues was significantly increased in the model group compared with the control group (Figure 1D,E). The expression of 15-LOX, LC3 Ⅰ, and LC3 Ⅱ in brain tissues of rats with epilepsy was attenuated by the administration of low-dose and high-dose ATS as well as by SV.

### 3.2. Brain Metabolomics Analysis

**Figure 2:**
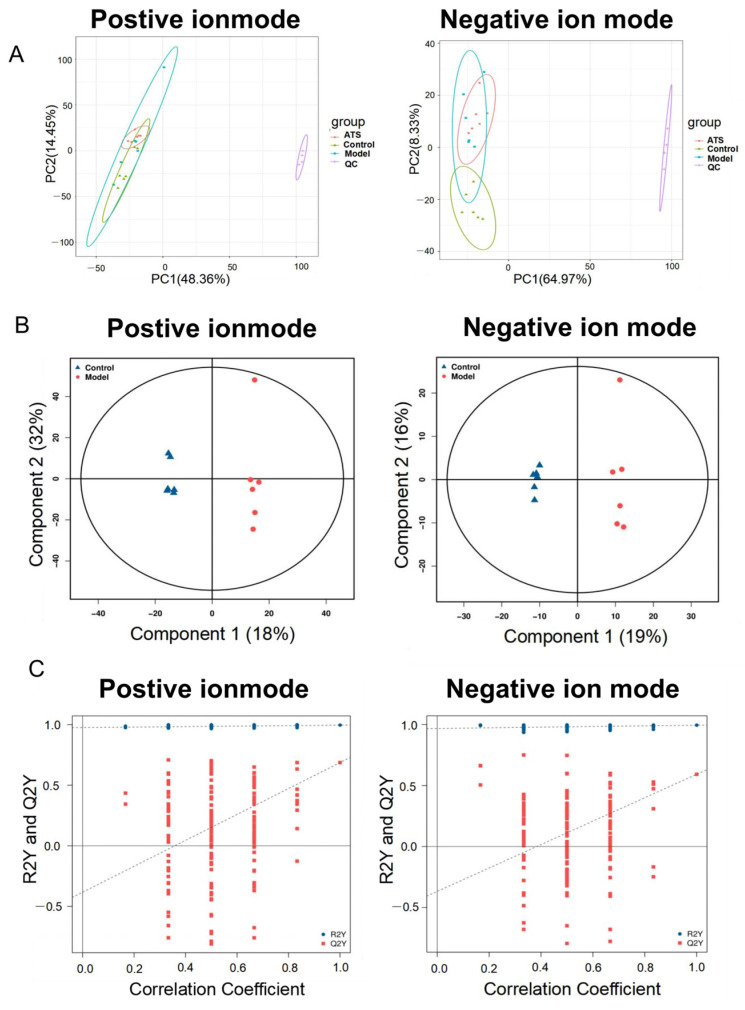
Multivariate statistical analyses of metabolomics data. (**A**) Positive and negative PCA score plots with QC. (**B**) Positive and negative OPLS-DA score plots between the control group and model group. (**C**) Positive and negative scatter plots of statistical validations obtained by 200× permutation tests.

The unsupervised principal component analysis (PCA) indicated tight clustering of all quality control (QC) samples, suggesting good robustness, stability, and repeatability of the method and analytical system [37]. Furthermore, there was a slight separation among the control, model, and ATS (50 mg/kg) groups in both ESI+ and ESI− modes (Figure 2A). OPLS-DA (orthogonal partial least squares discriminant analysis) is a statistical method used to model and predict group differences in multivariate datasets [38]. It helps in identifying patterns and variables that distinguish between different groups, making it a valuable tool for biomarker discovery. An OPLS-DA model was established to better elucidate between-group differences and further identify the metabolites differences of rats between the control and model groups (Figure 2B). The OPLS-DA model showed good predictive performance after 200 permutation tests and the regression curve of Q2 intersects negatively with the vertical axis, indicating its robustness without overfitting (Figure 2C).

### 3.3. Screen and Identification of Potential Biomarkers

**Figure 3:**
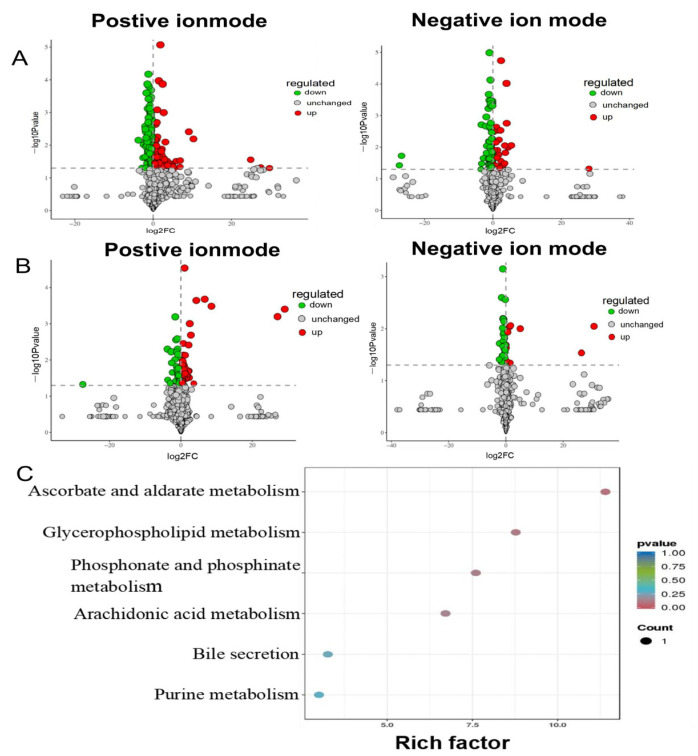
Pathway analysis for brain samples in PTZ-induced epileptic rats. (**A**) Positive and negative volcano plot between the control group and model group. (**B**) Positive and negative volcano plot between the model group and ATS group. (**C**) Distinct differential metabolites’ pathway enrichment bubble plot.

The OPLS-DA model was used to further explore the differential metabolites between different groups. VIP is a statistical measure used in multivariate analysis to identify the most influential variables contributing to the separation of groups in a dataset [39]. It is particularly useful in identifying key metabolites or features that drive differences between experimental groups. Then, metabolites with VIP score ≥ 1 and *p* < 0.05 were considered potential metabolic biomarkers. A total of 339 metabolites were identified as potential biomarkers between the model group and control group, including 211 metabolites in positive ion mode and 128 metabolites in negative ion mode, respectively (Figure 3A). Of these, 34 brain metabolites were significantly restored to control levels by ATS (Figure 3B). Twelve metabolites (hydroxymethyl cimetidine, succinoadenosine, morinidazole, allantoic acid, etc.) were upregulated, and 22 metabolites other than these were downregulated (Table 1). The metabolic pathways enrichment identified six epilepsy-related pathways, including ascorbate and aldarate metabolism, glycerophospholipid metabolism, phosphonate and phosphinate metabolism, arachidonic acid metabolism, bile secretion, and purine metabolism (Figure 3C).

### 3.4. The Operating Unit (OTU) of Gut Microbiota Classification and Diversity Analysis

**Figure 4:**
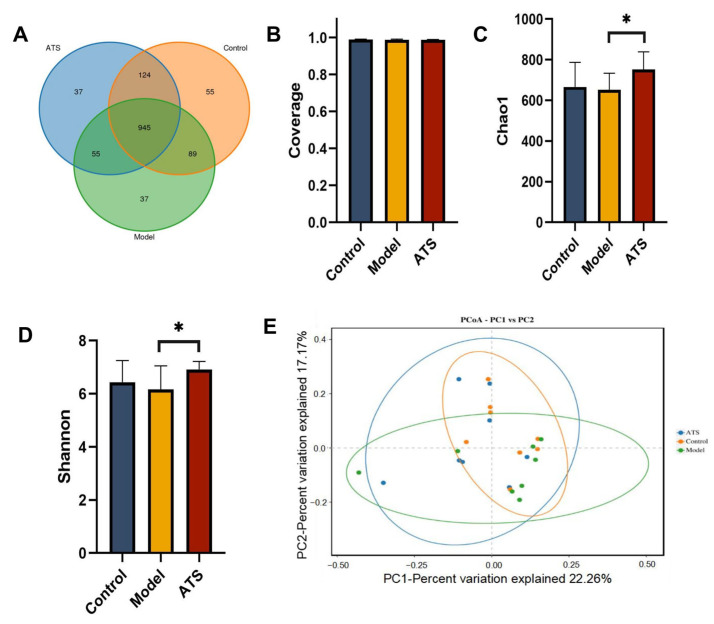
Effect of ATS on the α and β diversity of intestinal flora. (**A**) OTU expression in each group. (**B**) Sequencing depth of each group. (**C**) Chao 1 index. (**D**) Shannon index. (**E**) PCoA analysis of each group. * *p* < 0.05 vs. model group.

The 16S rRNA genes of fecal samples in the control group, model group and ATS group were amplified by PCR and then subjected to high-throughput sequencing. The OTU (Operational Taxonomic Unit) is used in microbiome studies to classify and compare microbial communities based on sequence similarity. It helps in understanding the diversity and composition of microbial populations. OTU analysis is a standard approach in microbiome research to identify and quantify different microbial taxa [40]. A total of 1342 OTUs were obtained (Figure 4A). The sequencing coverage indexes of the three groups were all greater than 0.99 (Figure 4B). These results indicated that the sample groups that were not tested had a low sequence probability. Compared with the control group, there was a certain degree of reduction in the Chao1 and Shannon indices for the model group (Figure 4C,D), and the abundance of gut microbiota had an uplifted tendency in the ATS group. Principal Coordinates Analysis (PCoA) is a method used to visualize the similarity or dissimilarity between samples based on their multivariate data [41]. The similarity of the intestinal flora of the samples (beta diversity) was also analyzed using PCoA analyses, which reduce the complexity of data without changing the main characteristics, thereby effectively extracting the most important features of the data. The result showed a certain separation trend between different groups, indicating that gut microbiota structure was different (Figure 4E).

### 3.5. Composition and Structure Analysis of Gut Microbiota

**Figure 5:**
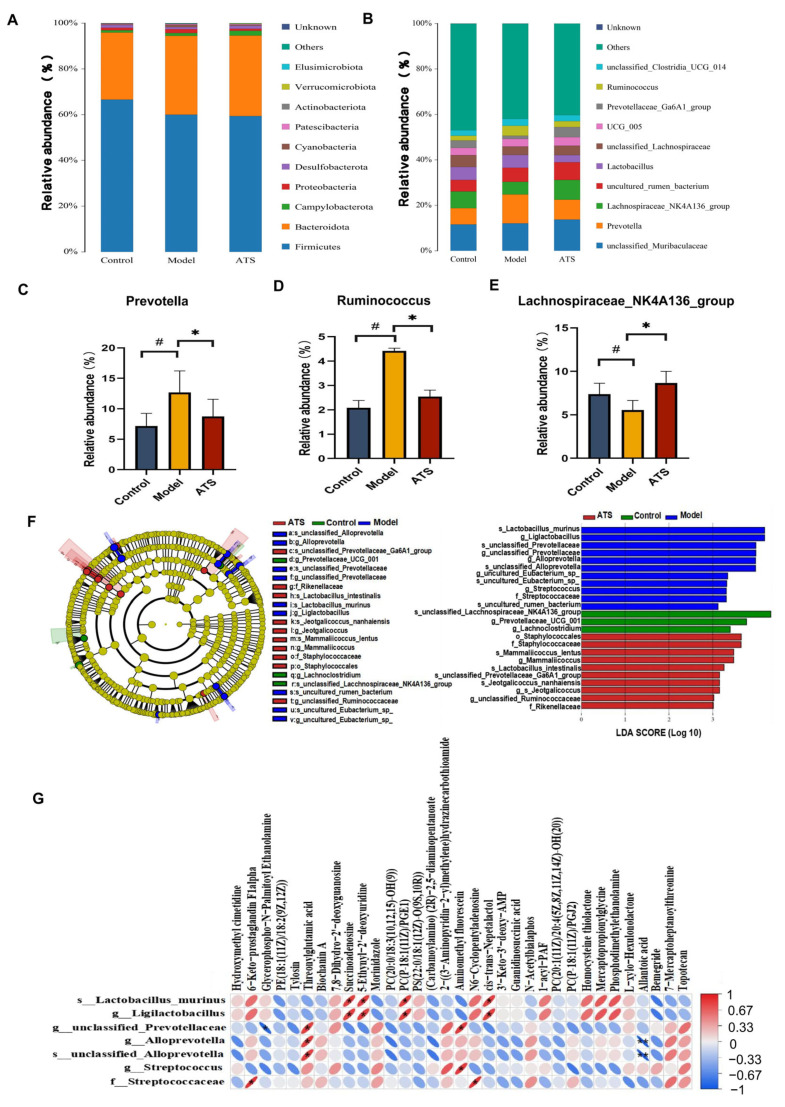
Effect of ATS on intestinal flora abundance and specific taxa. (**A**) Differences in the abundance of bacteria in each group at the phylum level. (**B**) Differences in the abundance of bacteria in each group at the genus level. (**C**) Relative abundance of Prevotella. (**D**) Relative abundance of Ruminococcus. (**E**) Relative abundance of Lachnospiraceae_NK4A136_group. (**F**) Expression of important flora in each group. (**G**) Correlations between the relative abundance of gut microbiota and metabolites. # *p* < 0.05 vs. control group, * *p* < 0.05 vs. model group.

Comparing the structural changes of intestinal flora in each group based on OTUs, at the phylum level (Figure 5A), Firmicutes, Bacteroidota, Campylobacterota, Proteobacteria, Desulfobacterota, Cyanobacteria, Patescibacteria, Actinobacteriota, Verrucomicrobiota, and Elusimicrobiota had higher abundances. The results indicate that these florae played important roles in each group. At the genus level (Figure 5B), compared with the model group, ATS mainly reversed Prevotella, Ruminococcus, and Lachnospiraceae_NK4A136_group. Among them, Prevotella and Ruminococcus were downregulated, and Lachnospiraceae_NK4A136_group was upregulated (Figure 5C–E).

LEfSe (Linear Discriminant Analysis Effect Size) is a method used to identify features (e.g., microbial taxa or metabolites) that significantly differ between groups [42]. It combines statistical significance with biological relevance to highlight important biomarkers. An LEfSe analysis was performed to further characterize the distinguishing phylotypes in the gut microbiota of the different groups, and the lower limit of the logarithmic LDA score was 3.0 (Figure 5F). The results showed that s_Lactobacillus_murinus, g_Ligilactobacillus, g_ Ligilactobacillus, g_unclassified_Prevotellaceae, g_Alloprevotella, s_unclassified_Alloprevotella, g_Streptococcus, and f_Streptococcaceae had higher expression in the epileptic rats. The Spearman correlation coefficient was used to calculate the correlation matrix level between intestinal dysbacteriosis and change in brain metabolites (Figure 5G). The results showed that there was a potential relationship among gut microbiota and brain metabolites during the development of epilepsy. For example, g_unclassified_Prevotellaceae was positively correlated with threonylglutamic acid but negatively correlated with glycerophospho-N-Palmitoyl Ethanolamine. The results of the study show that after PTZ-induced epilepsy in rats, the structure of the rat fecal flora underwent significant changes, and ATS could adjust or improve these changes. The results also show that intestinal flora plays an important role in the prevention and treatment of epilepsy.

## 4. Discussion

In the theory of TCM, ATS is a common aromatic drug for inducing resuscitation. It has been widely used in the treatment of brain diseases and nervous system diseases and has achieved satisfactory therapeutic effects [43]. However, its mechanism of action is not clear, which hinders the advancement of ATS antiepileptic research. Our study confirmed that oral ATS (50 mg/kg) significantly improved the seizure latency and pathology of rats with epilepsy. In our study, we used a dose of 50 mg/kg of ATS in mice, which is lower than the human-equivalent doses extrapolated from the recommended clinical range of 3–10 g/day for adults according to the *Chinese Pharmacopoeia* (2020 Edition). This highlights the need to carefully consider the dosing when translating our findings to human applications. This discrepancy underscores the challenges in directly translating animal doses to human applications. While our dose of 50 mg/kg has been shown to be effective in mice, it is important to recognize that human doses may need to be higher to achieve similar therapeutic effects. However, safety and potential toxicity must also be carefully considered when scaling up doses for human use. Future research should focus on dose optimization studies, pharmacokinetic and pharmacodynamic evaluations, and long-term safety assessments to better understand the therapeutic potential and appropriate dosing regimens of ATS in humans.

In this research, the mechanism behind the potential beneficial effects of ATS on epilepsy was investigated through intestinal microbiota and brain metabolomics methods. It was found that ATS can effectively relieve epilepsy through the microbiome–brain axis, deepening scientific understanding of epilepsy pathological development.

The metabolic pathways mediated by ATS mainly include four pathways: ascorbate and aldarate metabolism, glycerophospholipid metabolism, phosphonate and phosphinate metabolism, and arachidonic acid metabolism, which are closely linked to 34 potential biomarkers. These pathways play crucial roles in ATS’s therapeutic effects on PTZ-induced epilepsy. Research has found that ascorbic acid can regulate the release of glutamate (Glu) by heteromorphic conversion with Glu, thereby reducing the effect of Glu on neurons [44]. In in vitro experimental studies, ascorbic acid could regulate the redox of N-methyl-D-aspartate (NMDA) receptors, inhibit the activity of NMDA receptors, and block the binding of Glu to NMDA receptors [45]. Glu is an excitatory neurotransmitter that can cause seizures through receptor-mediated excitatory mechanisms [46]. As a Glu receptor, excessive activation of NMDA can lead to seizures [47]. Experimental evidence has shown that NMDA receptor antagonists can inhibit seizures [48]. The metabolism of arachidonic acid is a complex process involving multiple metabolic pathways and enzymes, mainly including the cyclooxygenase (COX) pathway, lipoxygenase (LOX) pathway, and cytochrome P450 pathway [49]. When epilepsy occurs, it can cause abnormalities in the arachidonic acid metabolism pathway [50]. For example, the neuroinflammation that causes epileptic seizures is related to the metabolite prostaglandin produced by the COX metabolism pathway [51]. While multiple testing corrections, such as Bonferroni or False Discovery Rate (FDR) corrections, were not applied in the current analysis, we acknowledge their importance in enhancing the validity of identified biomarkers. Future studies will incorporate these corrections to further validate the identified metabolites and reduce the likelihood of false-positive findings.

The potential application of specific microbiota as biomarkers and therapeutic targets is suggested by their close reflection of homeostasis and dysregulation in the brain and gut. According to the results of 16S rRNA sequencing, bacterial community abundance and diversity increased in ATS group rats compared to model group rats. Moreover, the β diversity showed a separation trend, indicating differences in gut microbiota structure between the ATS group and model group. At the genus level, ATS reverses three major intestinal microorganisms, namely Prevotella, Ruminococcus, and Lachnospiraceae_NK4A136_group. Prevotella can produce short-chain fatty acids, which play an important role in development of epilepsy [52], and low abundances of Ruminococcus and Lachnospiraceae_NK4A136_group could activate the parasympathetic nervous system and trigger a seizure [53]. Lachnospiraceae and Ruminococcaceae are known producers of butyrate, which has anti-inflammatory properties and helps maintain the integrity of the gut barrier [54]. The observed increase in Lachnospiraceae_NK4A136_group following ATS treatment suggests a potential mechanism by which ATS enhances antiepileptic action through the modulation of short-chain fatty acid (SCFA)-producing bacteria. Conducting a comprehensive metabolomic analysis to identify specific metabolites produced by the modulated microbiota will provide deeper insights into the metabolic pathways involved. This could include targeted analysis of SCFAs, bile acids, and other key metabolites.

The key microbial communities with significant differences between different groups were identified through LEfSe linear discriminant analysis. The results of correlation analysis showed that there was a potential relationship among gut microbiota and brain metabolites during the development of epilepsy. In epileptic rats, g_unclassified_Prevotellaceae was positively correlated with threonylglutamic acid and hyaluronan biosynthesis precursor 1, but negatively correlated with glycerophospho-N-Palmitoyl Ethanolamine. These results suggest that ATS exerts an antiepileptic effect by regulating the relative abundance of Prevotellaceae and by participating in the metabolism of glycerophospholipid metabolism.

The results suggest that ATS could mitigate PTZ-induced epilepsy. Ascorbate and aldarate metabolism, glycerophospholipid metabolism, arachidonic acid metabolism, and intestinal flora are crucial for ATS’s ability to counteract epilepsy. This study sheds light on how ATS diminishes PTZ-induced epilepsy through metabolomics and gut microbiota analysis, laying a groundwork for future investigations.

Nevertheless, the present study had some limitations. We only found that ATS can exert antiepileptic effects by regulating the gut microbiota and brain metabolites of PTZ-induced epileptic rats. Future research should use pseudo-germ-free model rats to explore the relationship between the microbiome and the metabolic spectrum and their interaction with epilepsy. Our study, while revealing promising antiepileptic effects of ATS in rat models, has limitations when considering translation to human epilepsy treatment. Species-specific differences in gut microbiota, brain metabolism, and epilepsy pathology between rats and humans may affect the applicability of our findings. Further research, including validation in human samples, is needed to address these limitations and ensure clinical relevance.

## 5. Conclusions

In conclusion, PTZ-induced epileptic seizures are closely associated with disruptions in brain metabolites and the gut microbiota. This study effectively validates the remarkable efficacy of ATS in treating epilepsy. ATS oral administration significantly increases seizure latency and alleviates pathological conditions in epileptic rats. This is achieved by regulating brain metabolic pathways and modulating the composition and function of the gut microbiota, thereby demonstrating its therapeutic potential in epilepsy treatment. This research not only lays the groundwork for further in-depth investigations into ATS but also highlights the potential of targeting intestinal microbes as a novel strategy for epilepsy treatment, providing a solid foundation for future studies exploring the therapeutic applications of ATS and the development of gut microbiota-based drug targets in epilepsy. However, these findings do not establish a direct causal link to epilepsy treatment. Future research should focus on mechanistic studies and controlled trials to validate the therapeutic potential of ATS in epilepsy and to explore the role of the gut microbiota as a novel target for epilepsy treatment.

## Figures and Tables

**Table 1:** Identified potential biomarkers regulated by ATS in positive and negative ion mode.

Mode	Metabolite	*m*/*z*	RT (min)	VIP	*p*-Value	Trend (Model vs. Control)	Trend (ATS vs. Model)
ESI−	Hydroxymethyl cimetidine	289.0869	2.3422	1.6938	0.0452	↓^#^	↑*
	6-Keto-prostaglandin F1alpha	369.2295	4.7634	2.2995	0.0025	↑^#^	↓*
	Glycerophospho-N-Palmitoyl Ethanolamine	452.2784	8.0968	1.6705	0.0409	↓^#^	↑*
	PE (18:1(11Z)/18:2 (9Z,12Z))	762.5077	9.6214	1.7988	0.0435	↑^#^	↓*
	Tylosin	950.4912	9.6714	2.0651	0.0065	↑^#^	↓*
	Threonylglutamic acid	247.0937	0.6684	1.8601	0.0222	↑^#^	↓*
	Biochanin A	319.0345	0.7034	1.7813	0.0326	↑^#^	↓*
	7,8-Dihydro-2′-deoxyguanosine	290.0868	0.8105	1.7534	0.0388	↑^#^	↓*
ESI+	Succinoadenosine	384.1176	1.8647	1.7709	0.0375	↑^#^	↓*
	5-Ethynyl-2′-deoxyuridine	252.0749	1.8718	1.8585	0.0266	↑^#^	↓*
	Morinidazole	271.1426	1.9575	1.7562	0.0429	↑^#^	↓*
	PC (20:0/18:3 (10,12,15)-OH(9))	810.6015	10.0276	1.8457	0.0337	↑^#^	↓*
	PC (P-18:1 (11Z)/PGE1)	824.5823	10.0347	1.7213	0.0315	↓^#^	↑*
	PS (22:0/18:1 (12Z)-O(9S,10R))	898.5612	11.1239	2.0329	0.0085	↑^#^	↓*
	(Carbamoylamino) (2R)-2,5-diaminopentanoate	155.0936	2.2209	2.3355	0.0050	↑^#^	↓*
	2-((3-Aminopyridin-2-yl) methylene) hydrazinecarbothioamide	413.1061	2.2209	1.7978	0.0473	↑^#^	↓*
	Aminomethyl fluorescein	362.1020	2.2637	1.9851	0.0222	↑^#^	↓*
	Hyaluronan biosynthesis, precursor 1	344.0949	3.0539	1.8754	0.0358	↑^#^	↓*
	N6-Cyclopentyladenosine	336.1668	3.6527	1.8747	0.0184	↑^#^	↓*
	cis-trans-Nepetalactol	191.1087	4.2366	1.7594	0.0332	↓^#^	↑*
	3′-Keto-3′-deoxy-AMP	387.0799	4.6784	1.7929	0.0311	↓^#^	↑*
	Guanidinosuccinic acid	389.0785	4.6784	1.8230	0.0264	↓^#^	↑*
	N-Acetylbialaphos	404.0991	6.5657	1.7645	0.0436	↑^#^	↓*
	1-acyl-PAF	538.3528	8.5529	1.8027	0.0274	↓^#^	↑*
	PC (20:1 (11Z)/20:4 (5Z,8Z,11Z,14Z)-OH (20))	816.5843	9.5429	1.8898	0.0271	↑^#^	↓*
	PC (P-18:1 (11Z)/PGJ2)	844.5398	9.9419	1.6700	0.0433	↑^#^	↓*
	Homocysteine thiolactone	100.0218	0.7177	1.9255	0.0240	↓^#^	↑*
	Mercaptopropionylglycine	146.0278	0.7177	1.9153	0.0210	↓^#^	↑*
	Phosphodimethylethanolamine	192.0340	0.7177	1.9254	0.0230	↓^#^	↑*
	L-xylo-Hexulonolactone	141.0195	0.7248	2.0583	0.0107	↓^#^	↑*
	Allantoic acid	199.0448	0.8533	1.8406	0.0236	↓^#^	↑*
	bemegride	155.0939	1.4086	2.1221	0.0120	↑^#^	↓*
	7-Mercaptoheptanoylthreonine	264.1257	1.7648	2.0653	0.0069	↑^#^	↓*
	Topotecan	444.1588	1.7933	1.8863	0.0171	↑^#^	↓*

# *p* < 0.05, vs. control group, * *p* < 0.05, vs. model group. ↑ Represents an increase and ↓ represents a decrease for the comparison of the model group vs. control group or the ATS (50 mg/kg) group vs. model group.

## Data Availability

The original data are available upon reasonable request from the corresponding authors.

## Supplementary Materials

The following supporting information can be downloaded at: https://www.mdpi.com/article/10.3390/biology14050488/s1, Figure S1: Western blot membrane of 15-LOX (~70 kDa) protein detected with anti-15-LOX (A6864; 1:1000; Abclonal, Wuhan, China) antibody; Figure S2: Western blot membrane of LC3 (~14 kDa) protein detected with anti-LC3 (14600-1-AP; 1:2000; Proteintech, Chicago, USA) antibody; Figure S3: Western blot membrane of β-actin (~41 kDa) protein detected with anti-β-actin (AC026; 1:50000; Abclonal, Wuhan, China) antibody.

## Abbreviations

The following abbreviations are used in this manuscript:

SV	Sodium valproate
PTZ	Pentylenetetrazol
ATS	*Acorus tatarinowii* Schott
PCA	Principal component analysis
OPLS-DA	Orthogonal projections to latent structures-discriminant analysis

## References

[1] Lu Y., Zhan R., Song B., Zhou Y., Zhu L., Chen H., Chen X. (2022). The optimized biocatalytic synthesis of (S)-methyl 2-chlorobutanoate by *Acinetobacter* sp. lipase. Chirality.

[2] Manole A.M., Sirbu C.A., Mititelu M.R., Vasiliu O., Lorusso L., Sirbu O.M., Ionita Radu F. (2023). State of the Art and Challenges in Epilepsy-A Narrative Review. J. Pers. Med..

[3] Zhou L., Gao Y., Lu H., Liu W., Xu X., Xing B., Liang X., Wang N., Jiang X., Zhao Q. (2023). Notopterygium incisum Root Extract (NRE) Alleviates Epileptiform Symptoms in PTZ-Induced Acute Seizure Mice. CNS Neurol. Disord. Drug Targets.

[4] Pong A.W., Xu K.J., Klein P. (2023). Recent advances in pharmacotherapy for epilepsy. Curr. Opin. Neurol..

[5] Pan I., LoPresti M.A., Clarke D.F., Lam S. (2020). The effectiveness of medical and surgical treatment for children with refractory epilepsy. Neurosurgery.

[6] López González F.J., Rodríguez Osorio X., Gil-Nagel Rein A., Carreño Martínez M., Serratosa Fernández J., Villanueva Haba V., Donaire Pedraza A.J., Mercadé Cerdá J.M. (2015). Drug-resistant epilepsy: Definition and treatment alternatives. Neurologia.

[7] Harward S.C., Rolston J.D., Englot D.J. (2020). Seizures in meningioma. Handb. Clin. Neurol..

[8] Maguire M., Singh J., Marson A. (2018). Epilepsy and psychosis: A practical approach. Pract. Neurol..

[9] Lin C.H., Hsieh C.L. (2021). Chinese herbal medicine for treating epilepsy. Front. Neurosci..

[10] Wang M., Tang H.P., Wang S., Hu W.J., Li J.Y., Yu A.Q., Bai Q.X., Yang B.Y., Kuang H.X. (2023). *Acorus tatarinowii* Schott: A review of its botany, traditional uses, phytochemistry, and pharmacology. Molecules.

[11] Xu Z., Zhou X., Hong X., Wang S., Wei J., Huang J., Ji L., Yang Y., Efferth T., Hong C. (2023). Essential oil of *Acorus tatarinowii* Schott inhibits neuroinflammation by suppressing NLRP3 inflammasome activation in 3×Tg-AD transgenic mice. Phytomedicine.

[12] Lee Y.C., Kao S.T., Cheng C.Y. (2020). *Acorus tatarinowii* Schott extract reduces cerebral edema caused by ischemia-reperfusion injury in rats: Involvement in regulation of astrocytic NKCC1/AQP4 and JNK/iNOS-mediated signaling. BMC Complement. Med. Ther..

[13] Umeda T., Sakai A., Shigemori K., Nakata K., Nakajima R., Yamana K., Tomiyama T. (2024). New value of *Acorus tatarinowii*/gramineus leaves as a dietary source for dementia prevention. Nutrients.

[14] Kim C.J., Kwak T.Y., Bae M.H., Shin H.K., Choi B.T. (2022). Therapeutic potential of active components from *Acorus gramineus* and *Acorus tatarinowii* in neurological disorders and their application in Korean medicine. J. Pharmacopunct..

[15] Cheng G., Wang X., Wang C., Zhang Q., Zhang Y. (2024). Understanding the molecular mechanisms of Acori Tatarinowii Rhizoma: Nardostahyos Radix et Rhizoma in epilepsy treatment using network pharmacology and molecular docking. Medicine.

[16] Wang Z., Wang Z., Lu T., Chen W., Yan W., Yuan K., Shi L., Liu X., Zhou X., Shi J. (2022). The microbiota-gut-brain axis in sleep disorders. Sleep. Med. Rev..

[17] Yue Q., Cai M., Xiao B., Zhan Q., Zeng C. (2022). The Microbiota-gut-brain axis and epilepsy. Cell. Mol. Neurobiol..

[18] Chen Y., Xu J., Chen Y. (2021). Regulation of neurotransmitters by the gut microbiota and effects on cognition in neurological disorders. Nutrients.

[19] Wang Y., Zhuo Z., Wang H. (2023). Epilepsy, gut microbiota, and circadian rhythm. Front. Neurol..

[20] Russo E. (2022). The gut microbiota as a biomarker in epilepsy. Neurobiol. Dis..

[21] Xu S., Li X., Zhang S., Qi C., Zhang Z., Ma R., Xiang L., Chen L., Zhu Y., Tang C. (2023). Oxidative stress gene expression, DNA methylation, and gut microbiota interaction trigger Crohn’s disease: A multi-omics Mendelian randomization study. BMC Med..

[22] Mossad O., Batut B., Yilmaz B., Dokalis N., Mezö C., Nent E., Nabavi L.S., Mayer M., Maron F.J.M., Buescher J.M. (2022). Gut microbiota drives age-related oxidative stress and mitochondrial damage in microglia via the metabolite N6-carboxymethyllysine. Nat. Neurosci..

[23] Wang Q., Yang Q., Liu X. (2023). The microbiota-gut-brain axis and neurodevelopmental disorders. Protein Cell.

[24] Deng Y., Zhou M., Wang J., Yao J., Yu J., Liu W., Wu L., Wang J., Gao R. (2021). Involvement of the microbiota-gut-brain axis in chronic restraint stress: Disturbances of the kynurenine metabolic pathway in both the gut and brain. Gut Microbes.

[25] Liu L., Wu Q., Chen Y., Ren H., Zhang Q., Yang H., Zhang W., Ding T., Wang S., Zhang Y. (2023). Gut microbiota in chronic pain: Novel insights into mechanisms and promising therapeutic strategies. Int. Immunopharmacol..

[26] Zhao Z., Ning J., Bao X.Q., Shang M., Ma J., Li G., Zhang D. (2021). Fecal microbiota transplantation protects rotenone-induced Parkinson’s disease mice via suppressing inflammation mediated by the lipopolysaccharide-TLR4 signaling pathway through the microbiota-gut-brain axis. Microbiome.

[27] Shearer J., Scantlebury M.H., Rho J.M., Tompkins T.A., Mu C. (2023). Intermittent vs continuous ketogenic diet: Impact on seizures, gut microbiota, and mitochondrial metabolism. Epilepsia.

[28] Mu C., Nikpoor N., Tompkins T.A., Choudhary A., Wang M., Marks W.N., Rho J.M., Scantlebury M.H., Shearer J. (2022). Targeted gut microbiota manipulation attenuates seizures in a model of infantile spasms syndrome. JCI Insight.

[29] Xu L., Chang C., Jiang P., Wei K., Zhang R., Jin Y., Zhao J., Xu L., Shi Y., Guo S. (2022). Metabolomics in rheumatoid arthritis: Advances and review. Front. Immunol..

[30] Lista S., González-Domínguez R., López-Ortiz S., González-Domínguez Á., Menéndez H., Martín-Hernández J., Lucia A., Emanuele E., Centonze D., Imbimbo B.P. (2023). Integrative metabolomics science in Alzheimer’s disease: Relevance and future perspectives. Ageing Res. Rev..

[31] Solch R.J., Aigbogun J.O., Voyiadjis A.G., Talkington G.M., Darensbourg R.M., O’Connell S., Pickett K.M., Perez S.R., Maraganore D.M. (2022). Mediterranean diet adherence, gut microbiota, and Alzheimer’s or Parkinson’s disease risk: A systematic review. J. Neurol. Sci..

[32] Sinclair E., Trivedi D.K., Sarkar D., Walton-Doyle C., Milne J., Kunath T., Rijs A.M., de Bie R.M.A., Goodacre R., Silverdale M. (2021). Metabolomics of sebum reveals lipid dysregulation in Parkinson’s disease. Nat. Commun..

[33] Zhang Y., Chen X., Mo X., Xiao R., Cheng Q., Wang H., Liu L., Xie P. (2023). Enterogenic metabolomics signatures of depression: What are the possibilities for the future. Expert. Rev. Proteom..

[34] Bastos de Araújo D., Gurgel do Amaral A.L., Maia da Fonseca S., Rodrigues de Souza K., Santos da Paz A.P., Jóia de Mello V., Barbosa G.B., Otake Hamoy M.K., Hamoy M. (2023). *Lippia origanoides* essential oil possesses anticonvulsant effect in pentylenetetrazol-induced seizures in rats: A behavioral, electroencephalographic, and electromyographic study. Front. Pharmacol..

[35] Chen L., Li J., Fang C., Wang J. (2025). Metabolomics-Based Study on the Anticonvulsant Mechanism of Acorus tatarinowii: GABA Transaminase Inhibition Alleviates PTZ-Induced Epilepsy in Rats. Metabolites.

[36] Chen L., Li J., Li Q., Sun Q. (2024). Hepatotoxicity Induced by Methyl Eugenol: Insights from Toxicokinetics, Metabolomics, and Gut Microbiota. Curr. Issues Mol. Biol..

[37] Ma C., Bi K., Zhang M., Su D., Fan X., Ji W., Wang C., Chen X. (2010). Toxicology effects of morning glory seed in rat: A metabonomic method for profiling of urine metabolic changes. J. Ethnopharmacol..

[38] How M.S., Hamid N., Liu Y., Kantono K., Oey I., Wang M. (2023). Using OPLS-DA to Fingerprint Key Free Amino and Fatty Acids in Understanding the Influence of High Pressure Processing in New Zealand Clams. Foods.

[39] Belmonte-Sánchez J.R., Romero-González R., Arrebola F.J., Vidal J.L.M., Garrido Frenich A. (2019). An Innovative Metabolomic Approach for Golden Rum Classification Combining Ultrahigh-Performance Liquid Chromatography-Orbitrap Mass Spectrometry and Chemometric Strategies. J. Agric. Food Chem..

[40] Zheng Q., Bartow-McKenney C., Meisel J.S., Grice E.A. (2018). HmmUFOtu: An HMM and phylogenetic placement based ultra-fast taxonomic assignment and OTU picking tool for microbiome amplicon sequencing studies. Genome Biol..

[41] Wang L.L., Zhang F.Y., Dong W.W., Wang C.-L., Liang X.-Y., Suo L.-L., Cheng J., Zhang M., Guo X.-S., Jiang P.-H. (2020). A novel approach for the forensic diagnosis of drowning by microbiological analysis with next-generation sequencing and unweighted UniFrac-based PCoA. Int. J. Leg. Med..

[42] Cao Q., Sun X., Rajesh K., Chalasani N., Gelow K., Katz B., Shah V.H., Sanyal A.J., Smirnova E. (2021). Effects of Rare Microbiome Taxa Filtering on Statistical Analysis. Front. Microbiol..

[43] Wang Z.J., Zhu Y.Y., Yi X., Zhou Z.S., He Y.J., Zhou Y., Qi Z.H., Jin D.N., Zhao L.X., Luo X.D. (2020). Bioguided isolation, identification and activity evaluation of antifungal compounds from *Acorus tatarinowii* Schott. J. Ethnopharmacol..

[44] Chiu Y.H., Wu Y.W., Hung J.I., Chen M.C. (2021). Epigallocatechin gallate/L-ascorbic acid-loaded poly-γ-glutamate microneedles with antioxidant, anti-inflammatory, and immunomodulatory effects for the treatment of atopic dermatitis. Acta Biomater..

[45] Liu W., Li Y., Zhao T., Gong M., Wang X., Zhang Y., Xu L., Li W., Li Y., Jia J. (2023). The role of N-methyl-D-aspartate glutamate receptors in Alzheimer’s disease: From pathophysiology to therapeutic approaches. Prog. Neurobiol..

[46] Kovács Z., Skatchkov S.N., Szabó Z., Qahtan S., Méndez-González M.P., Malpica-Nieves C.J., Eaton M.J., Kardos J., Héja L. (2022). Putrescine intensifies Glu/GABA exchange mechanism and promotes early termination of seizures. Int. J. Mol. Sci..

[47] Janicot R., Shao L.R., Stafstrom C.E. (2022). 2-deoxyglucose and β-hydroxybutyrate fail to attenuate seizures in the betamethasone-NMDA model of infantile spasms. Epilepsia Open.

[48] Wanleenuwat P., Suntharampillai N., Iwanowski P. (2020). Antibiotic-induced epileptic seizures: Mechanisms of action and clinical considerations. Seizure.

[49] Wang J., Zeng Y., Song J., Zhu M., Zhu G., Cai H., Chen C., Jin M., Song Y. (2023). Perturbation of arachidonic acid and glycerolipid metabolism promoted particulate matter-induced inflammatory responses in human bronchial epithelial cells. Ecotoxicol. Environ. Saf..

[50] Kuo Y.M., Lee Y.H. (2022). Epoxyeicosatrienoic acids and soluble epoxide hydrolase in physiology and diseases of the central nervous system. Chin. J. Physiol..

[51] Chen Y., Nagib M.M., Yasmen N., Sluter M.N., Littlejohn T.L., Yu Y., Jiang J. (2023). Neuroinflammatory mediators in acquired epilepsy: An update. Inflamm. Res..

[52] Li X., Wang Q., Wu D., Zhang D.W., Li S.C., Zhang S.W., Chen X., Li W. (2022). The effect of a novel anticonvulsant chemical Q808 on gut microbiota and hippocampus neurotransmitters in pentylenetetrazole-induced seizures in rats. BMC Neurosci..

[53] Wei S., Mai Y., Hu L., Zheng R., Zheng D., Chen W., Cai Y., Wang J. (2023). Altered gut microbiota in temporal lobe epilepsy with anxiety disorders. Front. Microbiol..

[54] Chakraborty P., Gamage H.K.A.H., Laird A.S. (2024). Butyrate as a potential therapeutic agent for neurodegenerative disorders. Neurochem. Int..

