# Supplementary material for: Antiepileptic Effects of Acorus tatarinowii Schott in a Rat Model of Epilepsy: Regulation of Metabolic Axes and Gut Microbiota
Source: Biology (Basel). 2025 Apr 29;14(5):488. doi: 10.3390/biology14050488 (PMC12108817; doi:10.3390/biology14050488)
Supplement: Supplementary file 1 [file biology-14-00488-s001.zip › biology-3574071-supplementary.pdf]

Supplementary Materials

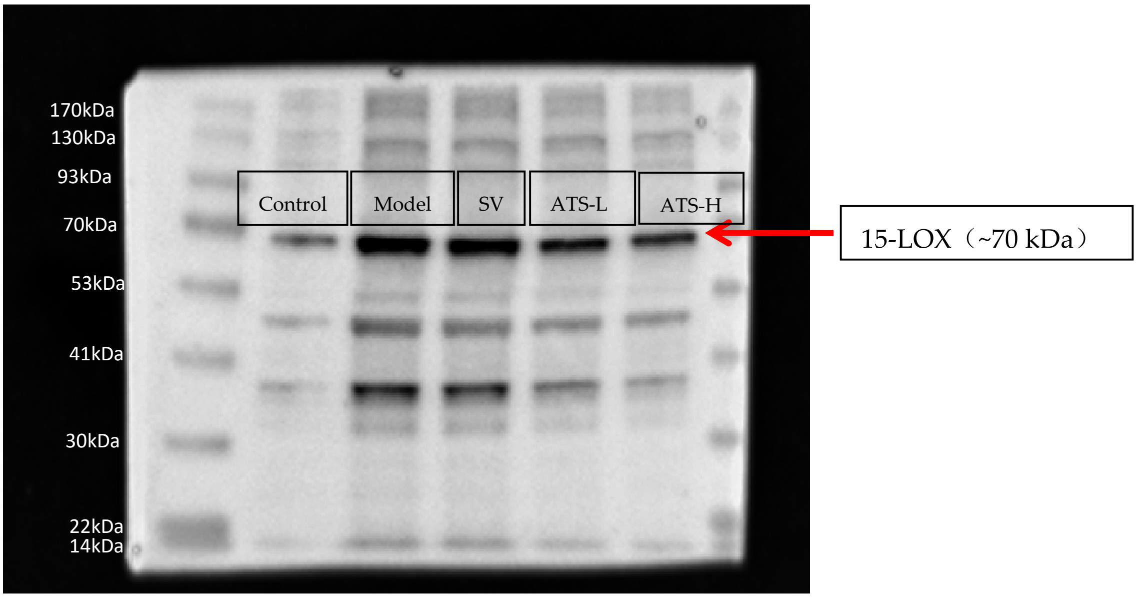

Figure S1. Western blot membrane of 15-LOX (~70 kDa) protein detected with anti-15-LOX (A6864; 1:1000; Abclonal, Wuhan, China) antibody.

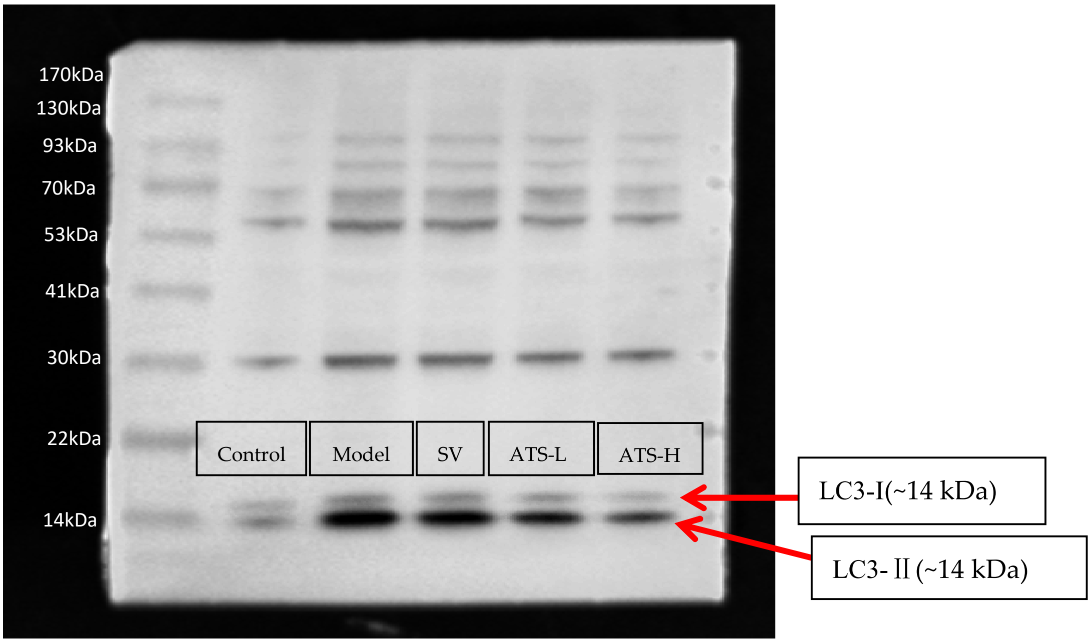

Figure S2. Western blot membrane of LC3 (~14 kDa) protein detected with anti-LC3 (14600-1-AP; 1:2000; Proteintech, Chicago, USA) antibody.

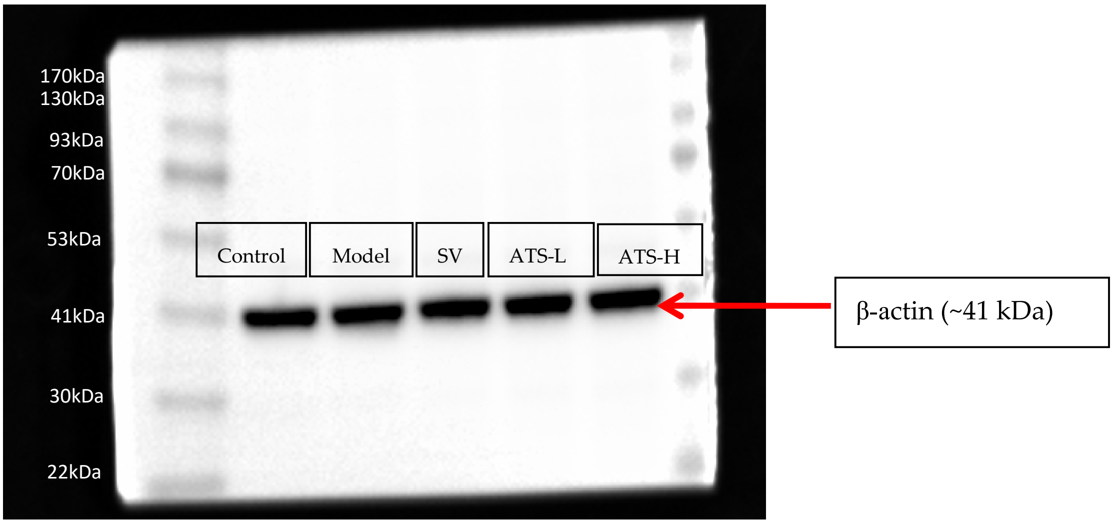

Figure S3. Western blot membrane of β-actin (~41 kDa) protein detected with anti-β-actin (AC026; 1:50000; Abclonal, Wuhan, China) antibody.
